# Altered Glymphatic System in Middle-Aged cART-Treated Patients With HIV: A Diffusion Tensor Imaging Study

**DOI:** 10.3389/fneur.2022.819594

**Published:** 2022-03-11

**Authors:** Benedictor Alexander Nguchu, Jing Zhao, Yanming Wang, Jean de Dieu Uwisengeyimana, Xiaoxiao Wang, Bensheng Qiu, Hongjun Li

**Affiliations:** ^1^Center for Biomedical Imaging, University of Science and Technology of China, Hefei, China; ^2^Department of Radiology, Beijing Youan Hospital, Capital Medical University, Beijing, China; ^3^School of Biological Science and Medical Engineering, Beihang University, Beijing, China

**Keywords:** HIV infection, HAND, glymphatic clearance function, ALPS index, diffusion tensor imaging, fluid homeostasis

## Abstract

**Objective:**

The brain relies on the glymphatic system to clear metabolic wastes and maintain brain homeostasis to fulfill its functions better. Yet, the complexity of the glymphatic flow and clearance and its changes in HIV infection and its role in neurocognitive dysfunction remain poorly understood. This study aims to explore the impact of HIV and combination antiretroviral therapy (cART) on the glymphatic system and establish a potential biomarker of HIV-associated neurocognitive disorders (HAND).

**Methods:**

Here, we examined the glymphatic profiles of middle-aged virosuppressed patients with HIV (*n* = 27) receiving cART over 1–6 years and healthy controls (*n* = 28) along the perivascular space (PVS) using diffusion tensor image analysis along the perivascular space (ALPS) with guided and unguided approaches. We later combined data from these analyses to investigate MRI glymphatic correlates of cognitive impairment and other clinical tests of HIV (CD4+ T-cell counts and CD4+/CD8+ ratio).

**Results:**

We found that glymphatic function as measured by the ALPS index increased significantly in the right and left PVSs of patients with HIV having cART. On antiretroviral therapy, a changing pattern in glymphatic clearance function in patients with HIV having cART correlated with attention and working memory. Duration on cART was also associated with cognitive performances of abstract and executive function and learning and memory.

**Conclusion:**

These findings provide MRI evidence of the presence of HIV-induced changes in the glymphatic flow and clearance, which might underlie cognitive impairment among patients with HIV having cART. An increase in the glymphatic activity might reflect a compensatory mechanism to regulate microenvironment homeostasis compromised by HIV. This compensation might be necessary to maintain the proper functioning of the brain while coping with HIV pathology. These findings also shed light on the clinical importance of evaluating glymphatic function based on the ALPS index and suggest that improving the glymphatic system may serve as an alternative therapeutic strategy for HAND.

## Introduction

Human immunodeficiency virus neuroinflammation has been associated with the pathogenesis of HIV-associated neurocognitive disorders (HAND) (1). Studies have shown that combination antiretroviral therapy (cART) can reduce these inflammations (2), enhance functional circuits (3, 4), and consequently improve cognitive functions (5). However, despite antiretroviral therapy, several studies have reported persisting cognitive dysfunction in patients with HIV having cART, especially in attention and working memory, executive function, motor control, and visual processing speed (6–8). We hypothesize that the disruption of homeostasis of the neural microenvironment, resulting from HIV-induced neurotoxins and elevated metabolites, may contribute to subsequent cognitive declines in HIV-infected individuals.

The regulation of fluid homeostasis in the brain is achieved through the glymphatic system. The glymphatic system refers to the drainage pathways through which metabolic waste products and other undesirable components get flushed out of the brain, thereby stabilizing microenvironmental homeostasis and providing suitable working conditions for the brain parenchymal cells (9, 10). The system relies on the bulk flow of cerebrospinal fluid (CSF) produced by the choroid plexus. The CSF-pressure and cerebrovascular pulsatility drive CSF into the deep para-arterial spaces where CSF-interstitial fluid (ISF) exchange occurs. CSF-ISF exchange is enabled by the polarization processes of aquaporin-4 (AQP4) water channels at the end-feet of astrocytes. This exchange facilitates the drainage of metabolic wastes and other soluble particles such as glucose, lipids, signaling molecules, and apolipoprotein E (apoE) *via* paravenous channels (11, 12).

Presently, the role that the glymphatic system plays in cART-treated HIV individuals is poorly known. Previous studies have documented the mechanisms of the glymphatic system and its roles in most neurodegenerative diseases (10, 13). An impairment in the glymphatic clearance function has been shown to accelerate the accumulation of abluminal protein deposits, α-synuclein, Aβ and tau, and huntingtin, which, respectively, underpin small vessel disease, Parkinson's disease, Alzheimer's disease (AD), and Huntington's disease (13, 14).

Studies have also hypothesized that the building up of these protein aggregates can reciprocally further impair glymphatic functioning (15). In a variety of research tests, impaired glymphatic clearance has been associated with cognitive dysfunction, with aged individuals being at higher risk due to age-related alterations such as weakening of arterial pulsatility and AQP4 polarization dysfunctions, and stiffness or flexibility of large elastic arteries (16). A recent work published in 2020 by Liu et al. provided evidence that restoring cognitive impairments was only possible when the underlying disruptions of the glymphatic system were normalized (10), suggesting a solid interdependence between the two (17).

Ways to enhance brain-to-blood clearance include promoting protein expressions for: (1) increasing CSF bulk flow; (2) stimulating Aβ phagocytosis; and (3) reducing microglia inflammatory activation (18–21). For example, osteopontin expressions promoted by glatiramer acetate immunization prove effective for macrophage-mediated Aβ clearance (20). Lipoprotein receptor-related protein 1 (LRP1) expressions promoted by omega-3 polyunsaturated fatty acids and lithium chloride can upgrade CSF-bulk flow and accelerate Aβ42-clearance (19, 21).

Whether there is a failure in the glymphatic functioning across patients with HIV that might contribute to HIV-associated cognitive decline among patients with HIV having cART remains unexplored. It is also yet to be determined what magnitude of glymphatic influx and clearance plays a pivotal role in cART strategies to reverse neural dysfunction. Here, using diffusion tensor imaging (DTI), we investigate whether the state of the glymphatic system in HIV-infected individuals provides additional prognostic information about the cause of HAND. To this end, middle-aged, virologically suppressed patients (age range, 36–55 years) were enrolled. The evaluation of glymphatic clearance functioning was performed using “DTI-analysis along the perivascular space” (DTI-ALPS) index. The DTI-ALPS index has proven effective for the estimation of glymphatic function in earlier studies (22–24). We hypothesize that the DTI-ALPS index would offer new insights into understanding the impact of HIV and cART on the glymphatic system and the role played by glymphatic clearance in the pathogenicity of HAND.

## Methods

### Participants

A total of 28 middle-aged healthy controls (mean ± SD age = 41.35 ± 4.85 years, range = 36–52 years) and 27 age-matched patients with HIV-1 seropositive taking cART [tenofovir (TDF) + lamivudine (3TC) + efavirenz (EFV)] (mean ± SD age = 40.30 ± 4.80 years, range = 36–53 years) were enrolled at Beijing YouAn Hospital, the capita Hospital, between March 2016 and November 2016. A set of clinical tests for blood and neuropsychological assessment were administered to these participants. The reports of the demographics, blood, and neuropsychological profiles are given in Table 1. Each patient sustained at least 1 year of stable cART-regimen. All the patients had undetectable viral loads (copies/ml). The blood test reports included CD4+ T-cell counts and CD4+/CD8+ ratio, which serve as potential indicators of HIV disease severity (25). The individuals who demonstrated any record of illicit drugs and alcohol use, cerebral atrophy, brain lesions, head injury, or neurological disorders were excluded from participation. This study was conducted in compliance with the code of Ethics of the World Medical Association (Declaration of Helsinki) for human experiments. Each participant's written informed consent was obtained before participation. The Ethical Committee of the Capital Medical University and the University of Science and Technology of China reviewed and approved this study.

**Table 1:** Demographic and clinical characteristics of patients with HIV and healthy controls.

**Category**	**HIV patients (*n* = 27)**	**Healthy controls (*n* = 28)**	***P*-value**
Age (mean ± SD years)	40.30 ± 4.80 (36–53)	41.35 ± 4.85 (36–52)	0.483
Gender (male %)	27 (100%)	28 (100%)	N/A
Duration on antiretroviral therapy (days) (IQR)	1119.82 ± 671.265 (365–2,342)	N/A	N/A
CD4+ cell count (cells/μl) (IOR)	503.15 ± 170.700 (148.0–804)	N/A	N/A
CD4+/CD8+ ratio (IQR)	0.72 ± 0.508 (0.15–2.09)	N/A	N/A
Undetectable viral load	27 (100%)	N/A	N/A
Learning and recall (memory) score	45.29 ± 8.85 (27.75–62)	N/A	N/A
Motor score	44.98 ± 13.26 (13–69)	N/A	N/A
Abstract/executive score	59.15 ± 9.73 (42.5–75.5)	N/A	N/A
Verbal and language score	48.30 ± 7.77 (36–60)	N/A	N/A
Attention/working memory score	47.43 ± 7.73 (37–77)	N/A	N/A
Information processing speed score	47.84 ± 8.36 (33–64)	N/A	N/A

*Data are shown in mean ± SD; IQR, interquartile range; N/A, not applicable; and p-values were computed based on a two-sample t-test*.

### Neuropsychological Testing

A battery of neuropsychological (NP) tests for six cognitive domains was administered to all patients according to Frascati recommendations (26). The Continuous Performance Test Identical Pairs (CPT-IP), the Wechsler Memory Scale-III (WMS-III), and Paced Auditory Serial Addition Test (PASAT) were used to assess attention and working memory. The category fluency and animal naming tests were used for testing verbal and language, the Grooved Pegboard test for motor function, and the Wisconsin Card Sorting Test-64 (WCST-64) for abstract and executive function. The Hopkins Verbal Learning Test-Revised (HVLT-R) and the Brief Visuospatial Memory Test-Revised (BVMT-R) tested for learning and recall, while the trail-making test part A evaluated the information processing speed. Demographically adjusted T-scores were obtained by standardizing the raw scores of each test. For the cognitive domain assessed by multiple tests, an average composite T-score was obtained across tests. NP test showing cognitive impairment in at least two cognitive domains with normal day-to-day functioning suggested ANI.

### Imaging Acquisition

Neuroimaging data were obtained using a Siemens 3T MRI Scanner (Allegra, Siemens Medical System, Erlangen, Germany) equipped with a 32-channel head coil. Imaging protocols include a 3D-T1-weighted anatomical image [TR/TE = 1,900 ms/2.52 ms, inversion time = 900 ms, flip angle = 9°, field of view (FVO) = 250 mm^2^ × 250 mm^2^, matrix size = 246 × 256, slice thickness = 1 mm, and voxel size =1 mm^3^ × 0.977 mm^3^ × 0.977 mm^3^] and a diffusion-weighted imaging [60 diffusion-encoded (b = 1,000 s/mm^2^), 3 references (b = 0 s/mm^2^), TR = 3,300 ms, TE = 90 ms, flip angle = 90°, slice thickness = 4.2 mm, voxel size = 2 mm^3^ × 2 mm^3^ × 4.2 mm^3^].

### Image Pre-processing

Data pre-processing was achieved using the FMRIB Software Library (FSL) (https://fsl.fmrib.ox.ac.uk/) (27), which involves correcting for susceptibility-induced distortions, eddy currents, and subject movements. Diffusion tensor maps were generated using the DTI-tensor fitting of the FSL. Other diffusivity maps, i.e., fractional anisotropy (FA) and mean diffusivity (MD), were also produced. Because all the neuroimaging data were collected from middle-aged adults, each subject's FA map was registered to the Illinois Institute of Technology (IIT) version 3.0 template of IIT Human Brain Atlas (28), using FMRIB's Linear/Non-linear Image Registration Tools (FLIRT/FNIRT), part of the FSL version 5.09 (27). The IIT v.3.0 template of the adult human brain is a population-based FA template having a high signal-to-noise ratio (SNR) and FA values and high image sharpness with visible small white matter structures and spatial features (28). It also has smaller intergroup FA differences and higher intersubject DTI spatial normalization accuracy compared with other DTI brain templates (28). Coregistration's accuracy was confirmed by visual inspection. Diffusion tensor maps of all of the subjects were normalized to IIT version 3.0 template using the transformation matrix obtained from the normalization of FA images to the IIT version 3.0 template.

### Glymphatic DTI-ALPS Analysis

**Figure 1:**
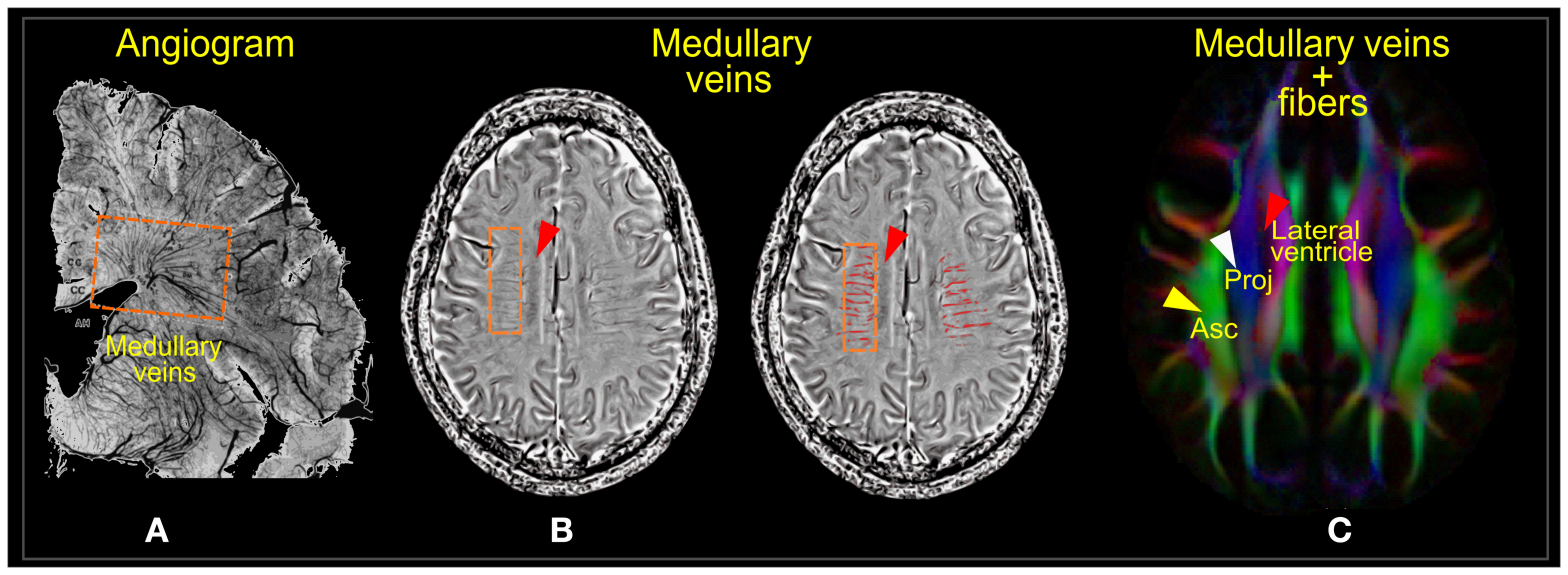
Medullary veins. **(A)** Angiogram of the parenchymal venous system. **(B)** Phase images of susceptibility-weighted imaging (SWI) showing the deep medullary venous structures before (left) and after segmentation (right). **(C)** Diffusion tensor image showing the projection (Proj) and association (Asc) fiber areas where the medullary veins run. Images **(A,B)** were adapted, with permission from Yan et al. (31).

**Figure 2:**
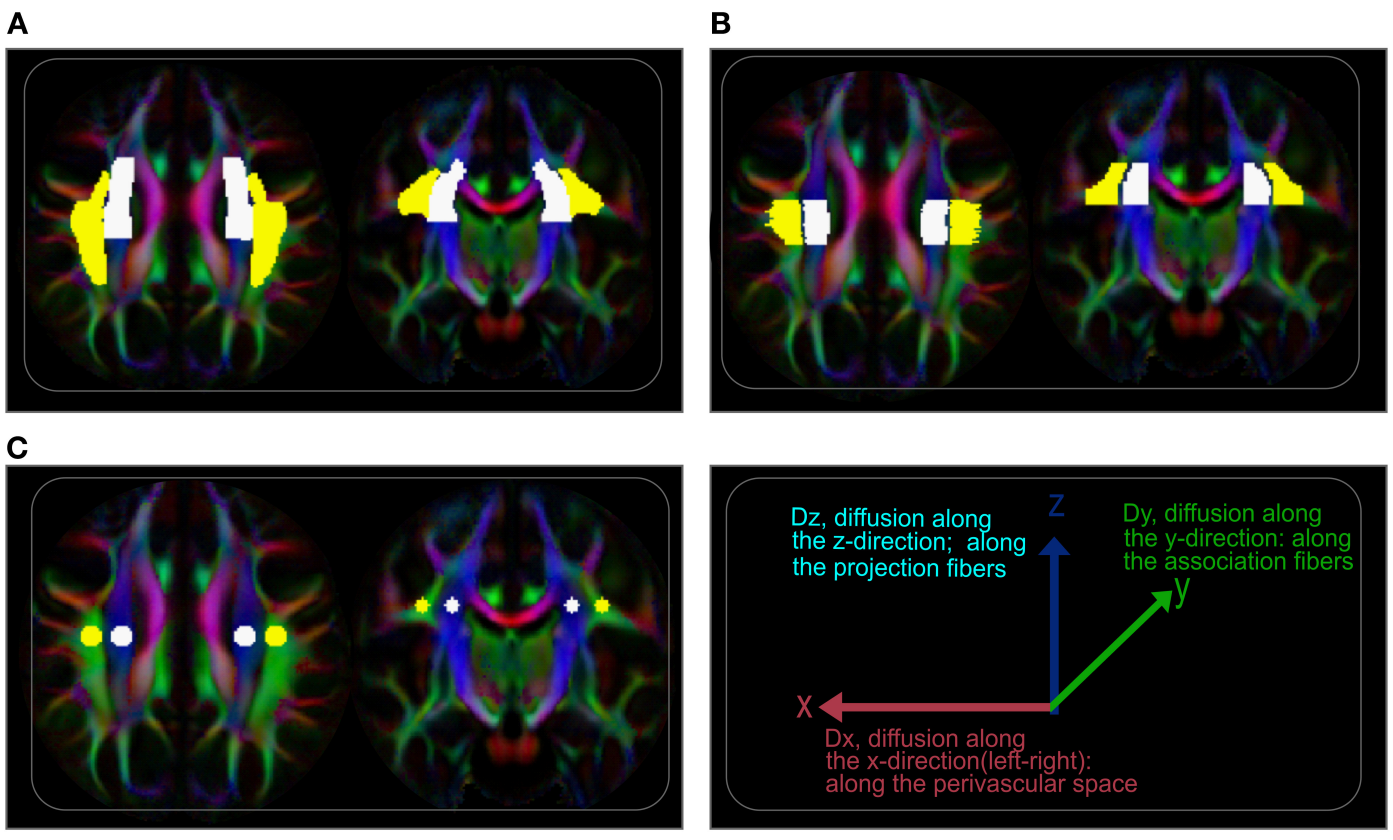
Regions of interest (ROIs) for ALPS index evaluation. **(A)** Axial (left) and coronal (right) visualization of the ROIs showing labels of the projection and association fiber areas defined by the labels of JHU-ICBM DTI-81 atlas. On the atlas-based approach (unguided), these labels were used to extract ALPS values from coregistered tensor maps. **(B)** Regions of the projection and association fiber areas along the periventricular space manually delineated, guided by diffusion maps (color-coded FA). **(C)** 5-mm-diameter spherical regions placed on the projection and association fiber areas with the centers on the reference slice.

Evaluation of diffusion tensor image analysis along the perivascular space index (DTI-ALPS-index) was concordant with earlier studies (24, 29, 30). We first registered JHU-ICBM DTI-81 labels, including the areas of projection fiber (superior corona radiata) and association fiber (superior longitudinal fasciculus), to IIT version 3.0 template. Then, DTI-ALPS index was computed as the estimate of water diffusivity along the x-axis of projection and association fiber areas (Dxpro, Dxasc) modulated by diffusivity along the y-axis of the projection and z-axis of the association fibers (Dypro, Dzasc), reflecting the glymphatic system in the medullary veins (Figure 1) (24, 32). The DTI-ALPS index is given by “mean (Dxpro, Dxasc)/mean (Dypro, Dzasc).” The DTI-ALPS index was calculated based on three paradigms. (1) Using the DTI-unguided atlas-based approach (Method 1), in which the regions of interest (ROIs) were set in the whole areas of the projection and association fibers as delineated by the JHU-ICBM DTI-81 Atlas (Figure 2A). (2) Using DTI-guided manually delineated ROIs (Method 2) of these fiber areas, with color-coded FA modulated by Illinois Institute of Technology (IIT) median eigenvector 1 (V1) (IITmedian_V1) used as reference (Figure 2B). (3) Using 5-mm-diameter spherical ROIs (Method 3) placed in the areas of the projection and association fibers, with the reference slice being the center of these ROIs (Figure 2C). Notice that the term DTI-guided refers to the use of FA color-coded maps to delineate the ROIs, while DTI-unguided means otherwise. The DTI-ALPS index was first evaluated separately for both the left and the right hemispheres at the locations where the direction of the deep medullary veins was perpendicular to the ventricle body. Then, the “bilateral DTI-ALPS index,” a joint DTI index of the right and left PVSs, was recorded.

For an intuitive understanding of the ALPS index at the ventricular level, it is essential to note the organization of the white matter (WM) fibers and their directions. The perivascular WM fibers (on the axial plane; Figure 1C) consist of two dominating fibers—the projection fibers (in the z-direction; blue; Figure 1C) and association fibers (in the y-direction; green; Figure 1C)—through which medullary veins run (Figure 1B). The direction of the PVS at the ventricular level is the direction of the medullary veins, which is also the x-direction (left/right or right/left), parallel to the walls of lateral ventricles but perpendicular to the projection and association fibers. Thus, the PVS can be well-described by differences in water molecule characteristics of the x-direction of the dominant fiber areas (Dxpro, Dxasc) and other perpendicular directions (Dypro, Dzasc) (22). This difference is mathematically presented as the ratio of the mean of the x-direction diffusivities [mean (Dxpro, Dxasc)] to the mean of the y- and z-direction diffusivities [mean (Dypro, Dzasc)].

### Statistical Analysis

Statistical analyses were performed on SPSS software [IBM SPSS Statistics for Windows, version 20.0 (IBM Corporation, Armonk, New York, USA)]. Group differences in the DTI-ALPS index were determined using the two-sample *t*-tests. Corrections for multiple comparisons were performed using a false discovery rate (*p* < 0.05, FDR). Correlation analyses between imaging markers and clinical measures were determined using Pearson's correlations.

## Results

### Demographic and Neuropsychological Evaluation

Table 1 shows the demographic and clinical data of participants. A total of 55 participants (HIV: 27, HC: 28, male: 100%) were included. The average age of patients with HIV having cART and healthy controls was 40.30 ± 4.80 and 41.35 ± 4.85 years old, respectively, which did not reach the significant level for the difference (*p* = 0.483: *p* > 0.05). The average duration on cART for patients with HIV was 1,119.82 ± 671.265 days. All the patients expressed an undetectable viral load. CD4+ cell counts (cells/μl) and CD4+/CD8+ ratios of patients were 503.15 ± 170.700 and 0.72 ± 0.508, respectively. Note that the reference ranges used for CD4 cell counts and CD4+/CD8+ ratios were 544 to 1,212 cells/μl and 0.71 to 2.78, respectively. The neuropsychological test scores of cognitive domains were: learning and recall: 45.29 ± 8.85, motor function: 44.98 ± 13.26, abstract and executive function: 59.15 ± 9.73, verbal and language: 48.30 ± 7.77, attention and working memory: 47.43 ± 7.73, and information processing speed: 47.84 ± 8.36. These neuropsychological test scores did not indicate detectable changes in cognitive functioning per Frascati criteria.

As **Figure 4** shows, duration of patients on cART was significantly related to cognitive scores of the domains of the abstract and executive function (*p* = 0.0009, *r* = 0.669, **Figure 4A**), and learning and recall (*p* = 0.0338, *r* = 0.444, **Figure 4B**). Neither CD4+ T-cell counts nor CD4+/CD8+ ratio demonstrated a significant relationship with the duration of patients on cART (*p* > 0.05).

### DTI-ALPS Index Group Differences

**Figure 3:**
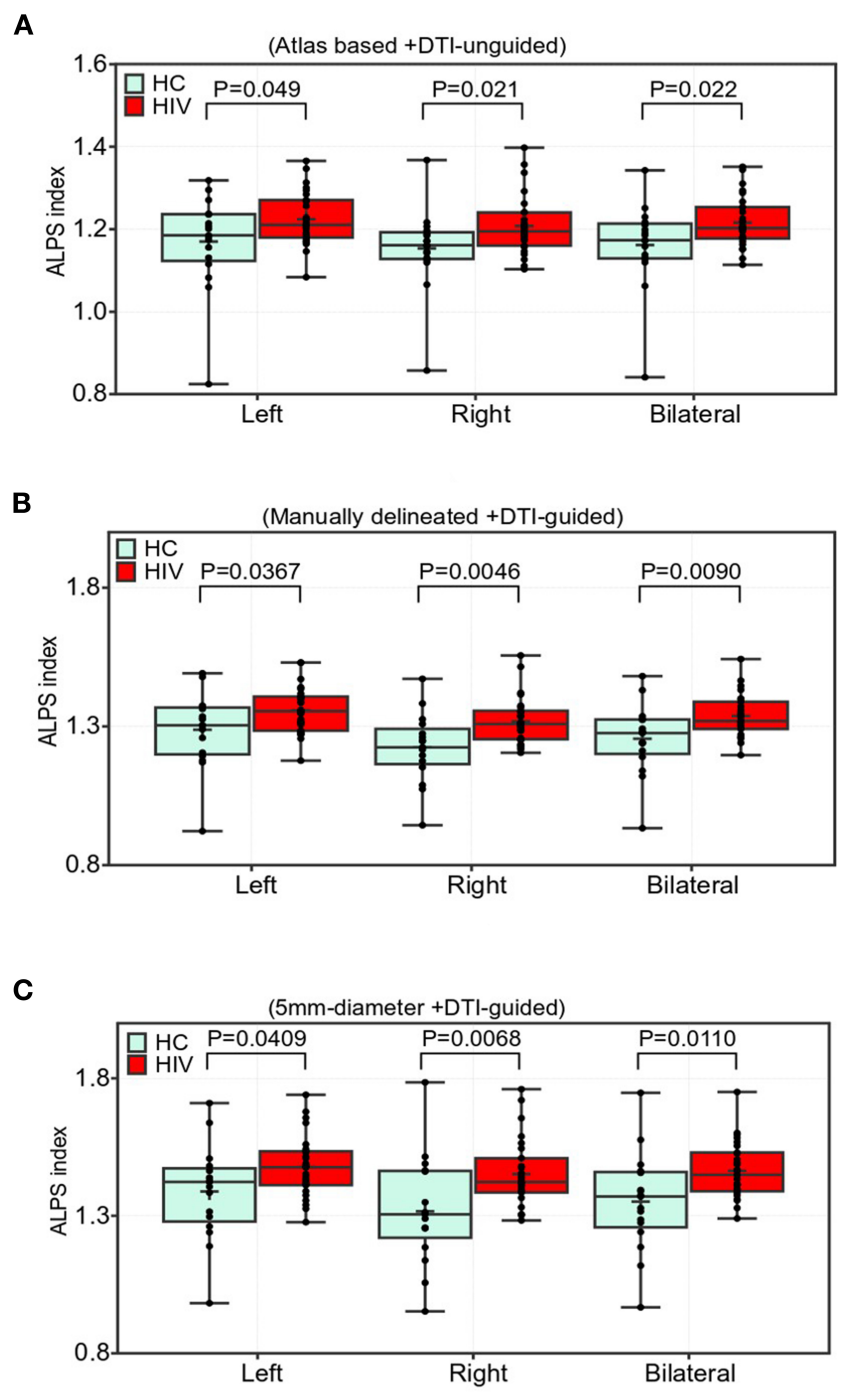
Comparisons of the ALPS indexes in the periventricular white matter (PVWM) veins between the HIV and HC groups. Two sample *t*-test with false discovery rate correction was used (FDR, *p* < 0.05). The group differences were based on **(A)** atlas-based approach, i.e., using labels from JHU-ICBM DTI-atlas and manual approaches using, **(B)** manually delineated regions of the lateral PVWM areas with reference to color-coded FA map, and **(C)** using 5-mm-diameter spherical ROIs. Participants in the cART-treated HIV group showed a significantly higher ALPS index than those in the HC group **(A–C)**. Boxes indicate the 25th−75th percentiles of the ALPS index, and the lines and whiskers show the median and range of the ALPS index, respectively.

Results of the group differences in the ALPS index are shown in Figure 3. All three paradigms of ALPS index evaluation reported significant group differences between patients with HIV having cART and healthy controls (HCs). Specifically, there was a significant increase in the ALPS index in the right, left, and bilateral PVSs of the HIV group (Figures 3A–C). The ALPS index computed using manually delineated guided-ROIs (Method 2, Figure 3B) offered the highest group difference with *p* = 0.0367, *t* = 2.16 (left); *p* = 0.0046, *t* = 2.99 (right); and *p* = 0.0090, *t* = 2.74 (bilateral), followed by 5-mm-diameter guided ROIs (Method 3, Figure 3C) with *p* = 0.0409, t = 2.11 (left); *p* = 0.0068, t = 2.85(right); and *p* = 0.0110, t = 2.66 (bilateral), and lastly the atlas-based DTI-unguided ROIs (Method 1, Figure 3A) with *p* = 0.0490, t = 2.03 (left); *p* = 0.0212, *t* = 2.12 (right); *p* = 0.0219, *t* = 2.19.

### Glymphatic Function and Cognitive Function

**Figure 4:**
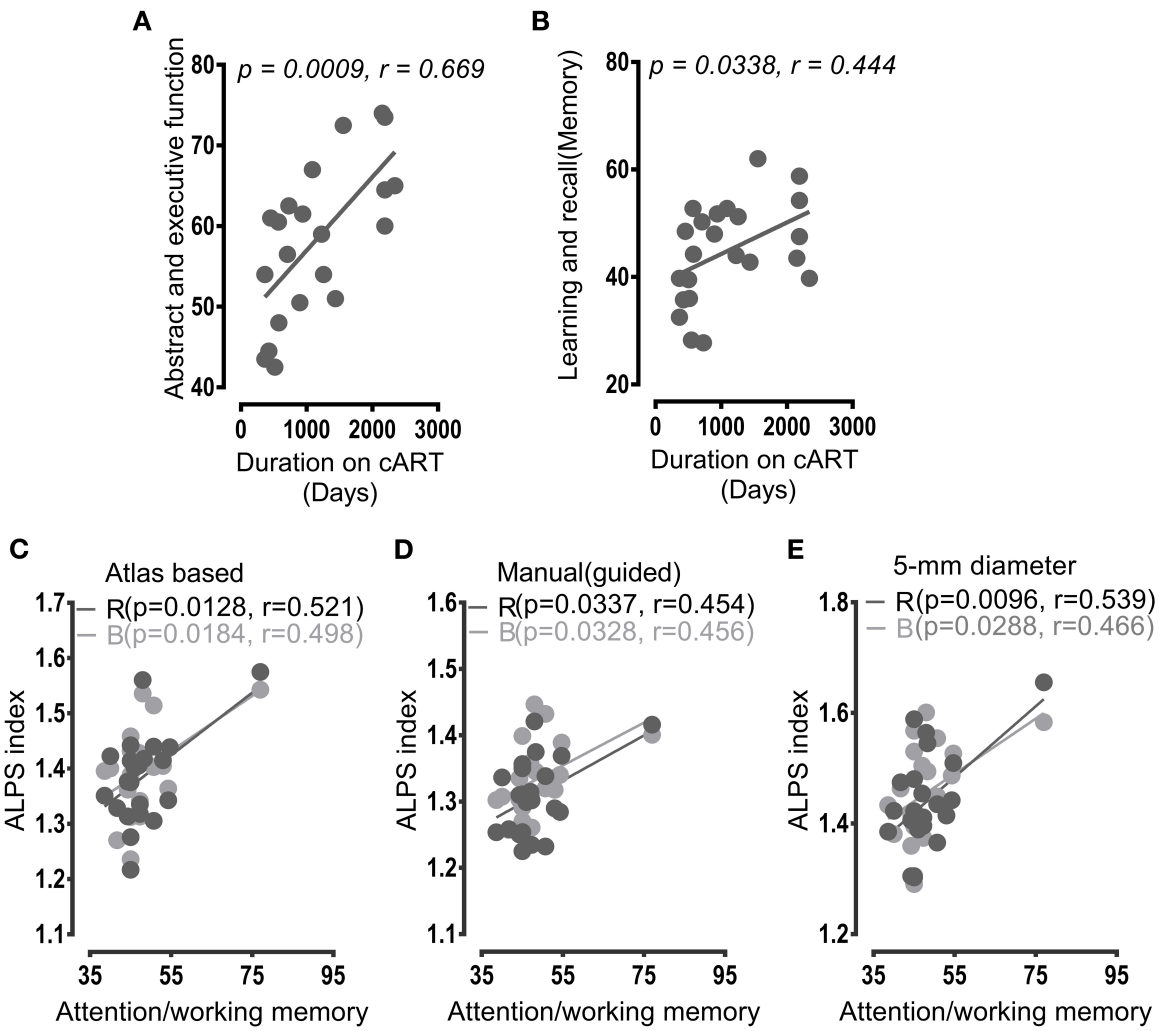
Correlation results. **(A,B)** Correlations of duration on cART with neuropsychological scores of patients with HIV. The duration of cART treatment was positively correlated with cognitive performances of **(A)** abstract and learning function and **(B)** learning and recall. **(C–E)** Correlations between the ALPS indexes and cognitive functions in HIV patients. **(C)** With JHU-ICBM DTI-81 atlas-based ROIs, the right (R, gray) and bilateral (B, green) ALPS indexes demonstrated positive correlations with attention and working memory. **(D)** With manual DTI-guided ROIs, positive relationships were also exhibited between attention/working memory and ALPS indexes of the right and the combined perivascular spaces (PVSs). **(E)** With 5-mm-diameter ROIs, similar correlations were manifested between the right and bilateral indexes and the attention and working memory neuropsychological scores. All tests in correlation analyses were significant at *p* < 0.05. R, right; B, bilateral. The term “bilateral index” refers to an index computed by combining ROIs of both sides (right and left) of the PVSs.

The results of correlations between the ALPS index and neuropsychological test scores are given in Figure 4. The diffusion characteristics, as measured by the DTI-ALPS index, along the PVWM were significantly associated with cognitive functions of cART-treated patients with HIV. The significant correlations were mainly between the ALPS index of the right and bilateral PVSs and attention and working memory (Figures 4C–E). From the DTI-unguided atlas-based approach (Method 1), the right ALPS index and bilateral ALPS index had significant correlations (*p* = 0.0128, *r* = 0.521; and *p* = 0.0184, *r* = 0.498, respectively) with attention and working memory (Figure 4C). From the manual whole-ROI DTI-guided approach (Method 2), the ALPS indexes of the right and bilateral reported respective associations (*p* = 0.0337, *r* = 0.454; and *p* = 0.0328, *r* = 0.456) with attention and working memory (Figure 4D). Similarly, the right and bilateral ALPS indexes of the 5-mm-ROI-based approach (Method 3) demonstrated significant associations with attention and working memory with *p* = 0.0096, *r* = 0.539; and *p* = 0.0288, *r* = 0.466 (Figure 4E). None of the other cognitive domains indicated significant correlations with the ALPS index.

### Duration on cART and DTI- Glymphatic Metric

**Figure 5:**
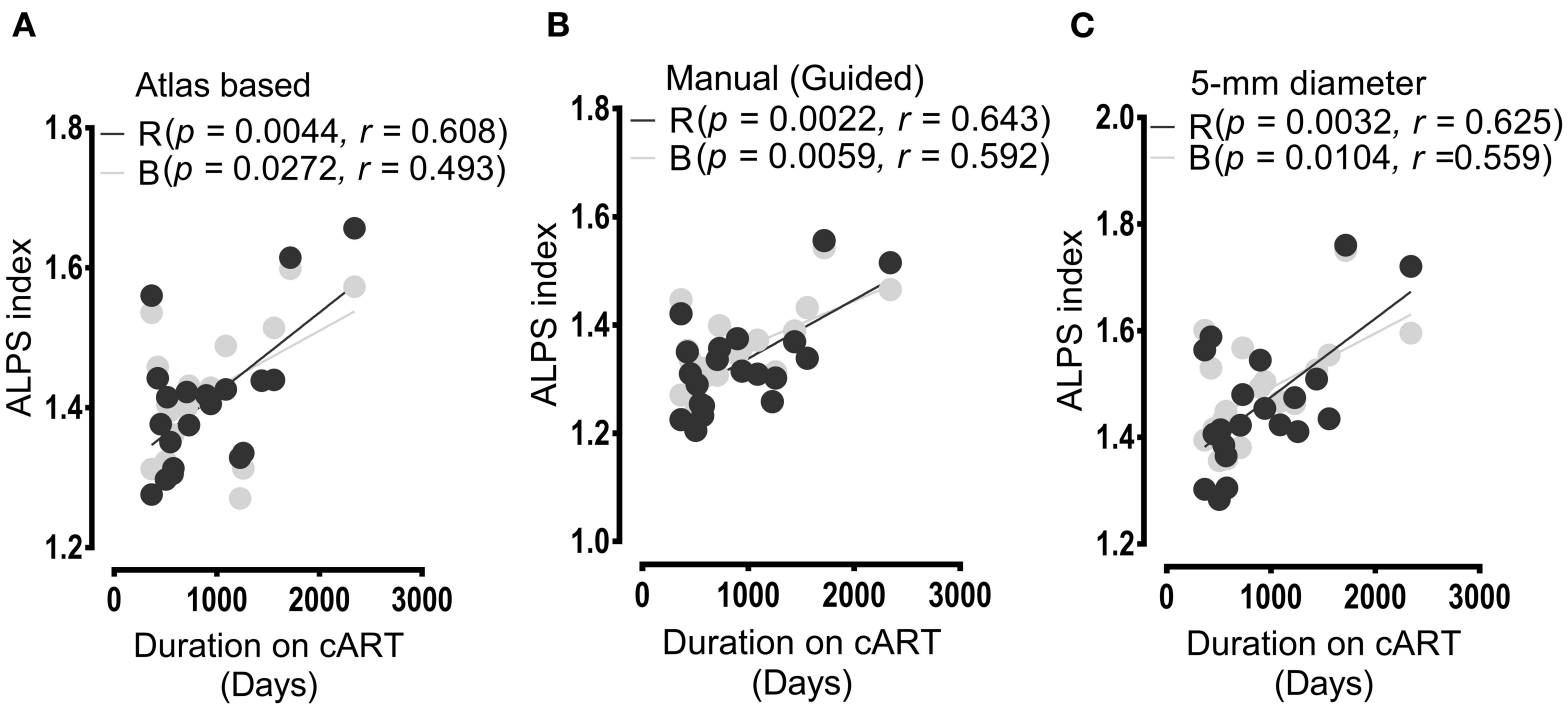
Results of correlations between the duration of cART treatment and ALPS index. In all analyses **(A,B)**, the duration of cART treatment correlated positively with the ALPS index of the right (R) and bilateral (B) PVSs. **(A)** Correlation outcomes as evaluated between the cART period and ALPS index computed within the areas defined by JHU-ICBM atlas-based ROIs. **(B)** Correlations as assessed between ALPS index calculated within areas determined by manual DTI-guided ROIs and duration of cART treatment. **(C)** Correlation outcome of the duration of therapy and ALPS index measured within areas of 5-mm diameter. All correlation results were significant at *p*< 0.05. In the figure legend, R, right; B, bilateral.

Figure 5 shows the results of the correlation between the duration on cART and ALPS index. The duration on cART exhibited a significant correlation with the ALPS index of the patients with HIV. As with cognitive score and ALPS index, the significant correlations of the duration on cART were predominantly with the ALPS index of the right and bilateral PVSs. The respective correlations scores were (*p* = 0.0044, *r* = 0.608, right) and (*p* = 0.0272, *r* = 0.493, bilateral) for ALPS indexes of Method 1 (Figure 5A); (*p* = 0.0022, *r* = 0.643, right) and (*p* = 0.0059, *r* = 0.592, bilateral) for ALPS indexes of Method 2 (Figure 5B) and (*p* = 0.0032, *r* = 0.625, right) and (*p* = 0.0104, *r* = 0.559, bilateral) for ALPS indexes of Method 3 (Figure 5C).

### DTI Glymphatic Characteristics and HIV-Immunologic Function

The glymphatic characteristics did not demonstrate any significant correlations (*p* > 0.05) with the HIV immunologic functions; that is, neither CD4+T-cell counts nor CD4+/CD8+ ratios were correlated with the ALPS index.

## Discussion

In this study, we evaluated the glymphatic system in cART-treated patients with HIV using the DTI-ALPS index, which is the ratio of diffusivity toward the PVS. First, we assessed the DTI-ALPS index with the directionality consistent with earlier studies (22), wherein the evaluation was conducted on the left sides of the participants' brains, which are considered to have thicker fibers as all subjects were right-handed. Second, this study added the evaluation of the right PVS, considering that patients with HIV are susceptible to HIV pathology in both hemispheres rather than one side (6, 33). In addition, we evaluated the average ALPS index of bilateral sides in line with Zhang et al. (24), which we referred to as bilateral ALPS index.

Herein, we found that the ALPS index of the right PVS showed a higher group difference than that of the left PVS, suggesting that a glymphatic change occurs differentially across PVSs, depending on the severity of HIV injury, adding to the point that a complete understanding of the neurobiology and status of glymphatic system requires an extensive assessment of both hemispherical PVSs than previously appreciated (22, 23). Nevertheless, we cannot exclude that other factors such as cART penetration might also account for the differences in glymphatic clearance function observed between the left and right sides. It is also worth noting that, of the three paradigms for evaluating glymphatic clearance function, method 2 and method 3 (DTI-guided approaches) offered higher statistical power than method 1 [DTI-unguided, also referred to as atlas-based method (30)]. This was expected since the DTI-unguided approach (Method 1) often incorporates diffusivities of water molecules in areas beyond the PVS (34), which could moderate the ALPS index values.

### Glymphatic Clearance Function and Fluid Homeostasis

Generally, the authors of this study have found that the ALPS index, as evaluated by all three paradigms, increased significantly in patients with HIV having cART compared to healthy controls. One possible interpretation is that the increase in the ALPS index suggests HIV-necessitated changes in glymphatic clearance functioning, possibly as a compensatory mechanism to maintain fluid homeostasis compromised by HIV.

**Figure 6:**
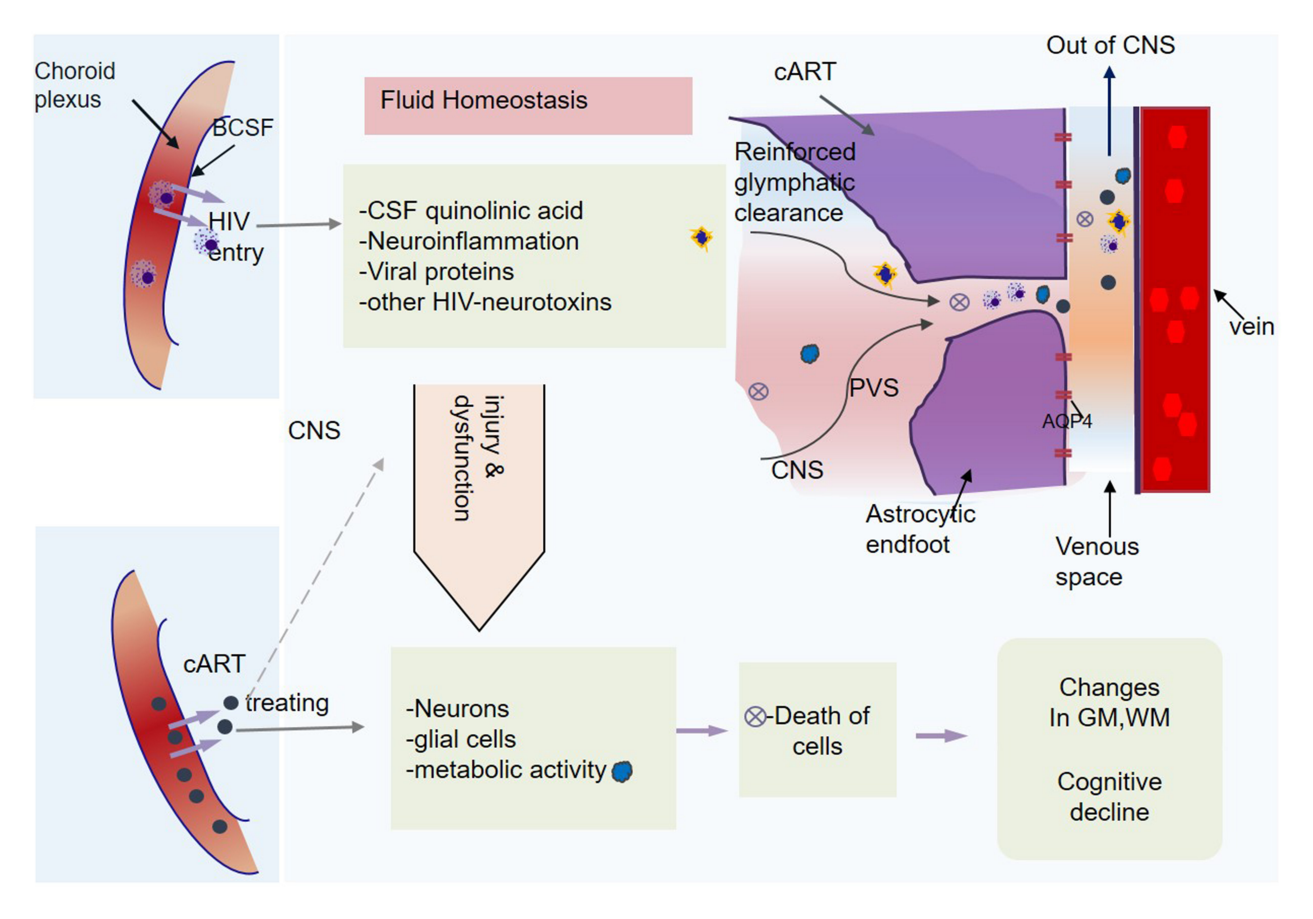
Central nervous system (CNS) homeostasis and glymphatic clearance. HIV elevates the cerebrospinal fluid (CSF) quinolinic acid levels. It also induces neuroinflammation, viral proteins that cause injury to neuronal and glial cells, including astrocytes, leading to the death of some cells. The metabolic activity and functions of CNS cells become subject to ongoing HIV injuries. In response to the toxic homeostasis and infectious chemicals, the brain might recruit higher-order mechanisms (AQP4 polarization processes, pulsatility, and cerebral vascular compliance) for homeostasis restoration.

Several previous studies have reported disruption of fluid homeostasis due to HIV (35, 36), wherein HIV has been shown to elevate levels of CSF quinolinic acids and induce abnormal microglial activation and neuroinflammation (also see Figure 6). These processes generate excess viral proteins in CNS, which attack neural and glial functions (35, 36) and metabolic regulatory system (37), and in the long run cause the death of the parenchymal cells (38). We hypothesize that such disruption of fluid homeostasis and the functional impairment of the glymphatic system might underlie cognitive deficits often seen among patients with HIV, especially in attention and working memory, executive function, and learning and memory (39, 40). Meanwhile, we also underscore that higher-order mechanisms of periventricular AQP4 polarization, arterial pulsatility, and cerebrovascular compliance play a valuable role in cognitively normal cART-treated patients with HIV to detox and cleanse the brain from HIV-associated neurotoxins and metabolites to maintain fluid milieu for normal functioning.

### Glymphatic Clearance Function in Antiretroviral Therapy

Our results differ from two earlier studies that examined the causes of cognitive impairment in patients with type 2 diabetes mellitus (23) or idiopathic normal pressure hydrocephalus (22) which found a lowered ALPS index. This may be explained by the fact that cART-treated patients with HIV in this study did not manifest observable cognitive changes in the neuropsychological battery tests, which may suggest that brain injury might occur early before deficits could be detected in the neuropsychological battery tests or might suggest stable cognitive reserve in patients with HIV despite HIV neuropathology, possibly favored by actions of the combination of [tenofovir (TDF) + lamivudine (3TC) + efavirenz (EFV)]. This is contrary to the patients (such as Alzheimer's, diabetic, or small vessel diseases) who are typically affected by constant accumulation and deposition of Aβ, which affects the ISF bulk flow (13), leading to a substantial decline of glymphatic functioning observed in these diseases (24).

Previous studies have demonstrated that cART can successfully penetrate the blood-cerebrospinal fluid barrier (BCSFB) and subsequently promote neutrophil and phagocytic activity, which is essential for the clearance of inflammatory immune complexes (41). The cART has also been shown to reduce the abnormal activity of viral proteins and glial activation, which normally have a deleterious effect on synoptodentritic structure and function (5, 42). So cART in our patients who have been on cART for over 1–6 years might have been protective against severe damage to cognitive reserve and thus allowed the brain to recruit other strategies—including glymphatic system, to cope with neuropathological damage due to HIV. Less protected CNS is at higher risk of developing PVS inflammation, vascular dysfunctions, and abnormal astrocytic AQP4 polarization, leading to later suppression of the glymphatic pathways (10), which collectively would weaken the brain capacity to modulate glymphatic clearance functioning. Moreover, it is essential to note that enhanced glymphatic functioning seen in our patients with HIV having cART could be imperative to clean the day-to-day remnants of the cART-drugs that crossed the CNS (43).

### Basal Ganglia, Glymphatic System, and HIV Infection

Earlier studies of the glymphatic system have already shown that the basal ganglia are entirely drained by deep medullary veins, indicating the more close relationship between the glymphatic system in the basal ganglia and the glymphatic clearance function calculated from the diffusion of PVSs around the deep medullary veins (24). The basal ganglia and the surrounding structures have also been reported to be the primary targets of HIV infection (7, 44–46). For example, following HIV infection, structural MRI studies have found tissues worn away in the structures of basal ganglia, including the caudate nucleus, and in the walls of lateral ventricles and the structures of the thalamus and corpus callosum (44–48). Learning and memory-related deficits in patients with HIV have been associated with injuries in these structures, including the caudate nucleus (49, 50). A study of white matter microstructures conducted in the USA in patients over 60 years old has also detected HIV injury in the dominant fibers (projection: corona radiata; and association: superior longitudinal fasciculus) crossed by these medullary veins, leading to abnormal diffusion and microstructural integrity (51).

Our results support these earlier findings, and provide further evidence of the drainage pathways of the lost tissues in the basal ganglia (see Figure 6) and other regions next to the medullary veins, which leads to reduction of basal ganglia and thalamic volumes (44, 52), expansion of the lateral ventricles (48), and thinning of the corpus callosum (46), which are usually detected in among patients with HIV. The authors of this study also infer that HIV injury might prolong to initiate infection in axons, which may further lead to abnormal flow or diffusion of water molecules in white matter tracts, and these changes may at least partly underpin changes in glymphatic clearance.

### Glymphatic System and Cognitive Function

The team of this study also found that increased glymphatic function in patients with HIV having cART was related to the cognitive function of attention and working. Previous studies have already documented the relationship between glymphatic function and cognitive function in patients with cerebral small vessel disease, diabetes, and AD (18, 22–24). Increasing activity or functions related to the cognitive function of attention and working memory in patients with HIV has previously been reported in a study performed by Chang et al., which investigated the neural correlates of attention and working memory deficits (53). The authors found that greater activation of the key brain regions such as frontal areas was required to perform more complex attention and working memory tasks, suggesting that neural correlates of attentional deficits due to HIV injury may be increased attentional modulation. Therefore, we infer that increasing glymphatic function in patients with HIV having cART might also reflect excessive modulation required for attention and working memory following HIV neuropathology.

Again, from correlation analyses, we also observed a significant correlation between the duration of cART treatment and cognitive performance of the patients with HIV. The longer the period on cART, the higher neuropsychological test scores of learning and memory and abstract and executive function. We speculate that this association may be mediated by cART. However, further studies are required to validate if this relationship is valid over the long term. This is because there is still an ongoing debate whether long-term use of cART has a detrimental effect on brain structure and function. For example, some studies have reported improvements in basal ganglia indicators in patients with HIV in the early use of cART, but later these improvements started to decline after the long-term use of cART, accompanied by cognitive deficits (54).

This study of middle-aged cART-treated patients with HIV did not find any relationship between glymphatic changes in the CNS compartments and HIV immunologic functions (i.e., CD4+T-cell counts and CD4+/CD8+ ratio), suggesting a generally healthy condition for these cART-treated individuals living with HIV (49).

## Limitations

This study has some limitations worth noting. First, all the analyses were performed using data acquired with b = 1,000 s/mm^2^ because: (1) water molecules with high motivity in PVSs are thought to have more significant influence in measurements when the b value is lower; (2) the signal-to-noise ratio in *b* = 1,000 s/mm^2^ is higher than in b = 2,000 s/mm^2^; and (3) the cognitive function has previously been demonstrated to correlate with ALPS index evaluated with b = 1,000 s/mm^2^ data than b = 1,000 s/mm^2^ data (23, 24, 29). However, the absence of comparisons with b = 2,000 s/mm^2^ results may limit our findings. Second, even though ALPS-index reflects the instantaneous glymphatic function at the time of the scan, adding the glymphatic evaluation at times of sleep demonstrated to have the more active glymphatic system (55) should be considered in further studies. It is also important to note that this study did not include untreated HIV cases. Furthermore, large, both gender, longitudinal data are warranted to validate conclusions of our findings.

## Conclusion

In conclusion, we found that the DTI-ALPS index in patients with HIV having cART was high. The higher ALPS index in cART-treated patients with HIV suggests enhanced glymphatic function. Moreover, the higher ALPS index was also associated with the cognitive scores of attention and working memory. Authors infer that HIV-neurotoxins disrupt the microenvironmental fluid homeostasis, which necessitates changes in glymphatic flow and clearance. Such changes in fluid homeostasis and glymphatic functioning might underlie HAND seen among patients with HIV having cART. These findings also suggest that the therapeutic initiatives aiming at improving functions of paravascular fluid exchange between CSF and ISF, cerebral arterial pulsatility, and perivascular AQP4 polarization would be the best strategies to maintain or normalize cognition despite the HIV pathology of an individual.

## Data Availability Statement

The raw data supporting the conclusions of this article will be made available by the authors, without undue reservation.

## Ethics Statement

The studies involving human participants were reviewed and approved by the Ethical Committee of the Capital Medical University and the University of Science and Technology of China. The patients/participants provided their written informed consent to participate in this study.

## Author Contributions

BN, JZ, YW, and XW: substantial contributions to conceptualization, methodology, data collection, image analysis, and writing—original draft preparation. BN, JZ, YW, JD, and HL: contributions to the data collection, image analysis, and interpretation of data. BN, XW, HL, and BQ: draft the work or review, edit, and revise it critically for important intellectual content. HL and BQ: final approval of the version submitted. All authors contributed to the article and approved the submitted version.

## Funding

This study was supported by the National Natural Science Foundation of China (grants 61936013, 81771806, and 21876041), the Ministry of Science and Technology of China (SQ2019YF013267), and the China Primary Health Care Foundation-Youan Foundation of Liver Disease and AIDS (BJYAYY-CY2019-04).

## Conflict of Interest

The authors declare that the research was conducted in the absence of any commercial or financial relationships that could be construed as a potential conflict of interest.

## Publisher's Note

All claims expressed in this article are solely those of the authors and do not necessarily represent those of their affiliated organizations, or those of the publisher, the editors and the reviewers. Any product that may be evaluated in this article, or claim that may be made by its manufacturer, is not guaranteed or endorsed by the publisher.
